# The Evolution of Body Size, Antennal Size and Host Use in Parasitoid Wasps (Hymenoptera: Chalcidoidea): A Phylogenetic Comparative Analysis

**DOI:** 10.1371/journal.pone.0078297

**Published:** 2013-10-14

**Authors:** Matthew R. E. Symonds, Mark A. Elgar

**Affiliations:** 1 Centre for Integrative Ecology, School of Life and Environmental Sciences, Deakin University, Burwood, Victoria, Australia; 2 Department of Zoology, University of Melbourne, Melbourne, Victoria, Australia; University of Arizona, United States of America

## Abstract

Chalcidoid wasps represent one of the most speciose superfamilies of animals known, with ca. 23,000 species described of which many are parasitoids. They are extremely diverse in body size, morphology and, among the parasitoids, insect hosts. Parasitic chalcidoids utilise a range of behavioural adaptations to facilitate exploitation of their diverse insect hosts, but how host use might influence the evolution of body size and morphology is not known in this group. We used a phylogenetic comparative analysis of 126 chalcidoid species to examine whether body size and antennal size showed evolutionary correlations with aspects of host use, including host breadth (specificity), host identity (orders of insects parasitized) and number of plant associates. Both morphological features and identity of exploited host orders show strong phylogenetic signal, but host breadth does not. Larger body size in these wasps was weakly associated with few plant genera, and with more specialised host use, and chalcidoid wasps that parasitize coleopteran hosts tend to be larger. Intriguingly, chalcidoid wasps that parasitize hemipteran hosts are both smaller in size in the case of those parasitizing the suborder Sternorrhyncha and have relatively larger antennae, particularly in those that parasitize other hemipteran suborders. These results suggest there are adaptations in chalcidoid wasps that are specifically associated with host detection and exploitation.

## Introduction

Developing parasitoids rely entirely on their host for nutrition, so the identity and range of their host species has a fundamental influence on the evolution of parasitoid life histories and reproductive strategies. For parasitoid species that exploit specific hosts, this antagonistic co-evolutionary process has resulted in diverse behavioural, physiological and morphological adaptations, including distinctive ovipositor shapes [1,2], specific egg-laying behaviours [3,4], and the ability to modify host behaviour [5].

Links between body size and host use have been reported in several parasitoids. Differences in body size within species may derive from differences in host species [6], host instars [7] and host sizes [8]. Differences in parasitoid size may have profound consequences on subsequent life history and fitness [3,9]. Likewise, inter-specific comparative studies reveal relationships between developmental stage of the host and parasitoid size [10], as well as between host species size and parasitoid species-average size [11].

The ability to detect and respond to host-derived chemical or auditory cues is another key adaptation of parasitoids [12]. Like most insects, the primary receptor structures that detect these cues are located on the antennae. Antennal morphology and antennal size may therefore vary within and across species in relation to aspects of host identity and biology. Both large-scale (antenna shape/size) and small-scale (sensilla type and arrangement) variation in antennal morphology are associated with host type and behaviour, and the exploitation behaviour of the parasitoid [13]. Chapman [14] suggested that insects that have highly specialised resource requirements (such as food sources, habitats or hosts) have a greater number of sensory receptors, thereby providing greater sensitivity and acuity towards chemical cues. Consistent with this prediction, recent studies comparing specialist and non-specialist species of braconid parasitoid wasps, revealed a greater abundance of chemosensillae and longer antennae [15,16] in the specialist species. However, it is not possible to ascertain whether these differences between the two species were due to increases in sensitivity *per se*, or simply a result of other differences between the species, such as overall body size. Other evidence for co-variation between antennal size and host species identity is reported for the solitary egg parasite *Telenomus alsophilae* Viereck, although the putative selective pressures driving these differences are unknown [17].

The chalcidoid wasps are among the most speciose superfamilies of insects, with roughly 23,000 species described and as many as 500,000 extant species estimated to exist, which are distributed globally [18]. Although some chalcidoids (such as the fig-wasps) are strictly phytophagous, their enormous diversity as a superfamily is related to the significant ecological and economic role they play as parasitoids of nearly all the insect orders (including Coleoptera, Diptera and other Hymenoptera). Individually, some species are highly specialised, relying on a single host species, whilst others are generalists, parasitizing dozens, if not hundreds, of species across several orders. Comparative investigations of the evolutionary significance of the diversity of chalcidoid life histories and morphological traits have been hampered by uncertain phylogenetic relationships [19]. Recent phylogenies for the group [18,20] now allow more rigorous investigations into the evolutionary processes that underlie this diversity.

Here we use morphological and ecological data from 126 chalcidoid wasp species to examine the correlation between host use and breadth, and body size and antennae size. Variation in antennal morphology in the Chalcidoidea is considerable [13], and forms an important part of taxonomic identification of species [21]. We test whether variation in body and antennal size is related to the taxonomic order of the species’ host. We also test Chapman’s [14] prediction that specialist parasitoid species have larger antennae (and hence potentially greater sensilla numbers) than generalist species. We also investigate the consequences of host plant associations for the parasitoid body size: plant associations, through their positive and negative effect on the herbivore’s capacity to resist parasitism, can have profound consequences for parasitoid fitness and body size [22]. Finally, we consider the role of life stage being parasitized. An earlier analysis [10] found that egg parasitoids tend to be smaller than species parasitizing other life stages. It may also be that antennal size differs if detection of eggs relies on different chemical cues compared with detecting larvae.

Because closely related species are likely to share traits due to their common ancestry, they do not necessarily provide statistically independent points for data analysis [23,24]. We conducted our analyses taking into consideration phylogenetic relationships using two recent extensive phylogenies for the group [18,20]. In addition to controlling for the effects of phylogeny, we examine whether there is strong phylogenetic signal in body and antenna size for parasitoids, and whether host breadth and parasitization of specific order of hosts is strongly predicted by phylogeny.

## Methods

### Chalcidoid data

We collated morphological data and host and plant association information for 126 species of Chalcidoidea from the Universal Chalcidoidea Database [25]. We used the extensive image gallery to make estimates of body size (body length) and antennal size (area) for each species. Images were uploaded into Image-J [26] for measurement estimation. Body size was taken as the length (in mm) of the straight line from the anterior edge of the head to the tip of the abdomen, not including ovipositor. Absolute estimation of this length was possible because all images have a scale bar on them. We also used only images where the wasp was in side-on, or top-down profile, to reduce the possibility of ‘shortening’ of body length as a result of the image being taken at an oblique angle. Antennal surface area was the area (mm^2^) of the polygon drawn around the antennae in clearest view on the picture. Because there is sometimes pronounced sexual dimorphism in body size and antennal size in parasitoid wasps [27,28], we used only data from female wasps, since their antennae and body size are more likely to be directly influenced by host identity. To conform with statistical assumptions of normality, body size and antenna size measures were log transformed for the analysis.

We estimated host breadth and diversity of plant associates as the number of species reported as primary hosts and the number of plant genera reported to be associated with the parasitoid species (as listed in the Universal Chalcidoidea Database). The distribution of the number of host species was strongly right-skewed (a small number of species are associated with >100 host species). Consequently the number of host species was log transformed before analysis to better conform with statistical assumptions of normality. We also counted the number of orders of hosts exploited, because the number of species alone may not reflect true host breadth if those species are all closely related [29]. For a broader characterisation, we classified wasps as firstly either parasitoid or non-parasitoid and, for the former category, either specialist (less than 10 species in a single order parasitized) or generalist (more than 10 species OR more than one order parasitized). Likewise we noted what stage of the host life-cycle was parasitized, i.e. whether the wasp was an egg parasitoid (=1) or parasitoid of larvae, nymphs or adults (=0). For each parasitoid species we created categorical (0/1) variables that noted whether they parasitized individual orders (=1) or not (=0). The orders most commonly exploited by chalcidoid wasps included Coleoptera, Diptera, Hemiptera, Hymenoptera and Lepidoptera. In the case of Hemiptera we also split the analysis into two subgroups with very different biologies, those species that parasitize the sessile Sternorrhyncha (aphids, whiteflies and scale insects), and those that parasitize the other hemipteran sub-orders such as Auchenorrhyncha and Heteroptera (leafhoppers, cicadas and true bugs). It is worthwhile to note that the majority of species (7 out of 9) that parasitize these other hemipteran suborders are also egg parasitoids (and conversely that 7 out of the 20 egg parasitoids in the analysis parasitize these other hemiptera). The complete data are provided as Table S1.

### Chalcidoid phylogeny

Phylogenetic topology (i.e. the hypothesised pattern of evolutionary relationships) can influence the conclusions of comparative analysis [24,30]. Consequently, in circumstances where there are competing phylogenies for a group, insight can be gained by carrying out analyses using more than one phylogeny [31], to see whether results obtained are qualitatively consistent. In the case of Chalcidoidea there are two recent extensive phylogenies available, both of which we used as the basis for analysis. The first of these is a molecular phylogeny based on 18S and 28S ribosomal gene regions for almost 700 species of Chalcidoidea produced by Munro et al. [18]. The second phylogeny is a combined analysis by Heraty et al. [20] based on a subset of 300 of the same taxa, using the same molecular data and alignment, but with the inclusion of 233 morphological characters. The phylogenies were pruned to include only the species in our analysis. For genera where these phylogenies do not provide resolution to the specific level we resolved inter-specific relationship as follows: Where the genus had only two species represented in our analysis, it was assumed the two species were sibling species with a branching point half way along the branch. For genera with more species represented we derived relationships from other published phylogenies: Triapitsyn et al [32] for relationships within *Anagyrus*; Guerrieri and Noyes [33] for relationships within *Metaphycus* (implied from taxonomic discussion); Auger-Rozenburg et al. [34] for relationships within *Megastigmus*; Heraty et al. [35] for relationships within *Aphelinus*; Darling and Cardinal [36] for relationships within *Leucospis*, and; Graham and Gijswijt [37] for relationships within *Torymus*. We also augmented our phylogenies with additional information on genera missing from the Munro et al. [18] and Heraty et al. [20] phylogenies using the following sources: Owen et al. [38] for resolution of the positions of *Megaphragma* and *Prestwichia*; Lotfalizadeh et al. [39] for the position of *Risbecoma*; Burks et al [40] for the position of *Aulogymnus* and *Horismenus*, and; Rasplus et al [41] for the position of *Seres* and *Apocrypta*. Remaining missing species from the Heraty et al. [20] phylogeny were filled in from the Munro et al. phylogeny [18]

**Figure 1:**
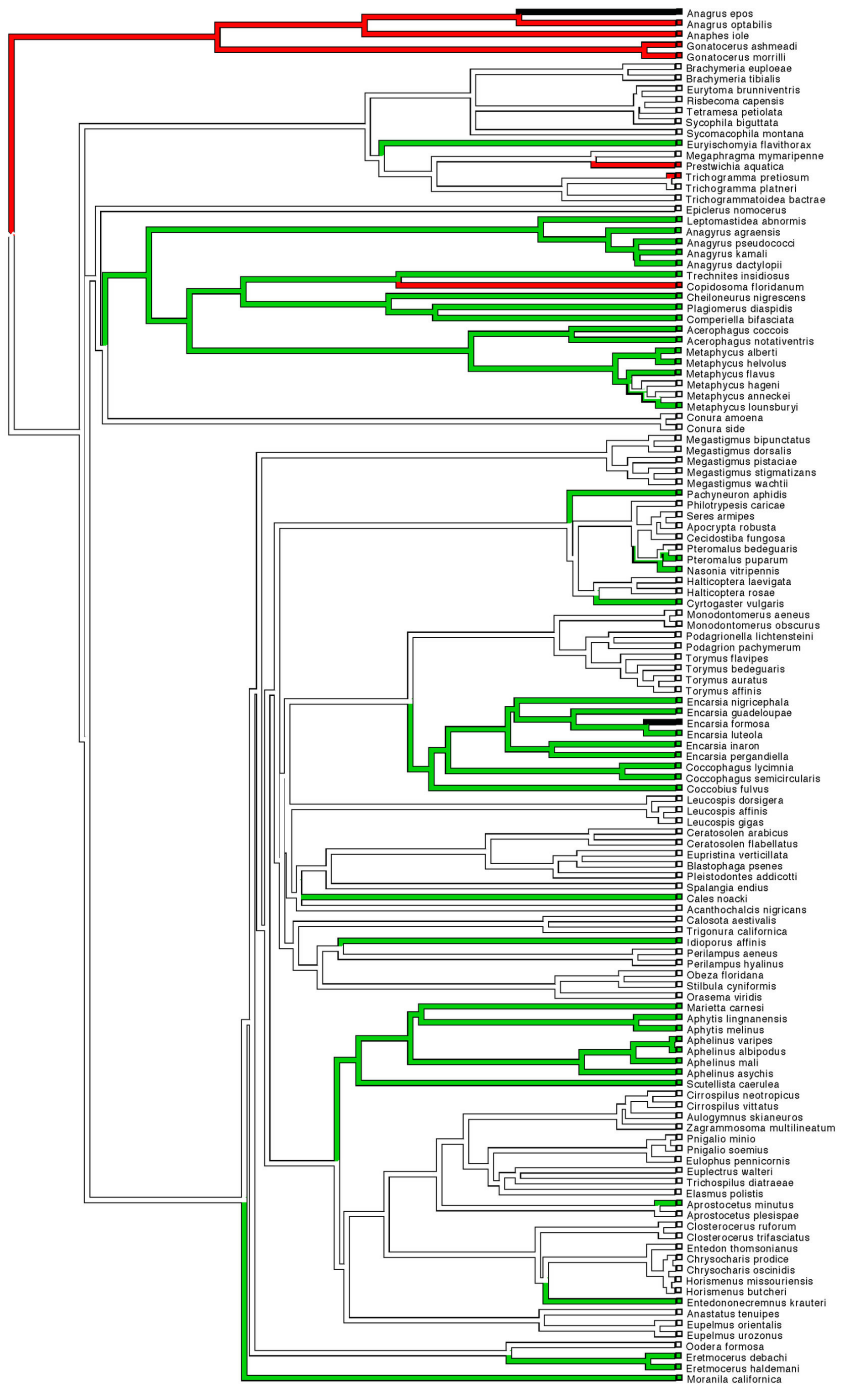
Chalcidoid phylogeny adapted from Munro et al [18]. First phylogeny used in the analysis based on Munro et al [18] of the 126 chalcidoid species used in the analysis indicating species parasitizing Sternorrhyncha (green), Hemiptera other than Sternorrhyncha (red), and both (black). Putative reconstruction of evolutionary transitions in based on maximum parsimony analysis in Mesquite [78].

**Figure 2:**
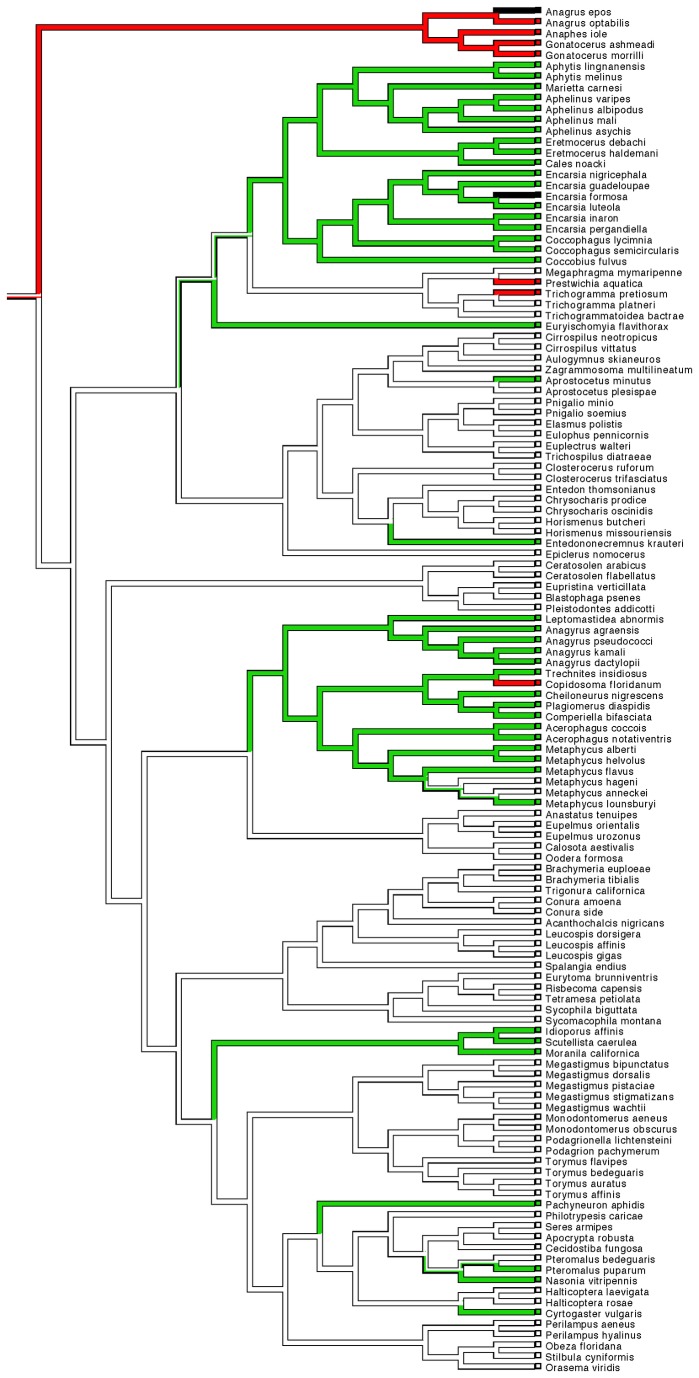
Chalcidoid phylogeny adapted from Heraty et al [20]. Second phylogeny used in the analysis based on Heraty et al [20] RAxML phylogeny produced using combined morphological and molecular data. Colouring and reconstruction of evolutionary transitions as per Figure 1.

For the Munro et al. [18] phylogeny, branch lengths were taken from the original phylogeny, with branch lengths for the additional intra-generic branches re-scaled from their source publications to fit this scheme. For a given species, *I*, that appears in both the Munro et al. phylogeny and the intra-generic phylogeny, the distance from the basal node to the species *I* tip was set to be the same in the latter phylogeny as the former. Using this as a reference point, the branch lengths to the remaining species in the intrageneric phylogeny could be recalculated on the same scale as the Munro et al. phylogeny. In cases where no branch length information was available from the source phylogeny, all branches were set to equal length. The final wasp phylogeny was ultrametricised using the penalized likelihood method [42], in order to conform with assumptions of the phylogenetic comparative analysis and phylogenetic signal analysis. For the Heraty et al. [20] phylogeny, branch length data were not available for the combined morphological and molecular tree, consequently equal branch lengths were assumed before ultrametricisation. The phylogenies used are presented in Figures 1 and 2.

### Comparative analyses

All phylogenetic comparative analyses were carried out using the *caper* [43], *ape* [44] and *geiger* [45] packages in R [46].

We first calculated phylogenetic signal, a quantitative measure of how much variation in a trait is related to phylogenetic relatedness. We used two separate metrics for this, depending upon the type of data.

For continuous variables (body size, antenna size, number of host species, number of host orders, and number of plant associates), we calculated Pagel’s λ [47,48]. λ is defined as the multiplier of the off-diagonal elements of the variance-covariance matrix of a trait (or traits) given the phylogenetic relationships of the species under test. The maximum likelihood value of λ is calculated as being the value that makes the expected variance-covariance matrix best fit the actual data. In simple terms, λ usually adopts a value between 0 and 1, where λ = 0 indicates no association between the trait and phylogeny (i.e. there is no tendency for closely-related species to have similar trait values), and λ = 1 indicates that the species’ traits covary exactly in the manner expected by a Brownian motion model of evolution (i.e. closely related species have very similar trait values). We also tested whether the calculated value of λ differed significantly from λ = 0 (i.e. whether there was any significant phylogenetic signal in the trait of interest)

For categorical variables (parasitoid vs. non-parasitoid, egg parasitoid vs. other species, specialist vs. generalist, species associations with particular orders of host), we used the *D* metric of Fritz and Purvis [49]. Basically, D compares the number of observed changes in the state of a binary categorical trait with the number expected under a Brownian motion model of evolution that produces the same number of tip species with each character state as the observed pattern (see Fritz and Purvis [44], for details of the exact calculation). It too generates a value between 0 and 1, although, in contrast to λ, a D value of 1 means that the trait has evolved in essentially a random manner (i.e. no phylogenetic signal), and a D value of 0 indicates the trait is highly correlated with phylogeny. Again, we estimated whether the calculated values of D differed significantly from 1 (no phylogenetic signal).

To test the relationships of body size and antenna size to host use/breadth and number of plant associations, we used phylogenetic generalized least squares (PGLS) regression [50]. The morphological parameters were our response variable, with host/plant variables set as predictor variables in separate models (PGLS is formulated for continuous variables, but can happily accommodate categorical predictor variables). For all models we calculated Akaike’s Information Criterion (AIC) [51], and compared the value obtained with that obtained for the null intercept-only model. As a general rule of thumb [51,52], if models are less than two AIC units better (lower) than the null model, then they cannot be considered distinguishable from a null model. Models between 2 and 6 AIC units better than the null model are considered probably better (but without ruling out entirely the null model), and more than 6 units better is considered very strong evidence in support of the model. To evaluate how much more likely each of our models were than the null model, we calculated the Evidence Ratio (ER) (see 52).

Because antennal size is correlated with body size, in all models predicting antenna size we also included body size as a predictor variable (to control for its effect). In the calculation of phylogenetic signal, for antennal size we used residual antenna size (the y-residual from the PGLS regression line).

## Results

### Antennae size and body size

**Figure 3:**
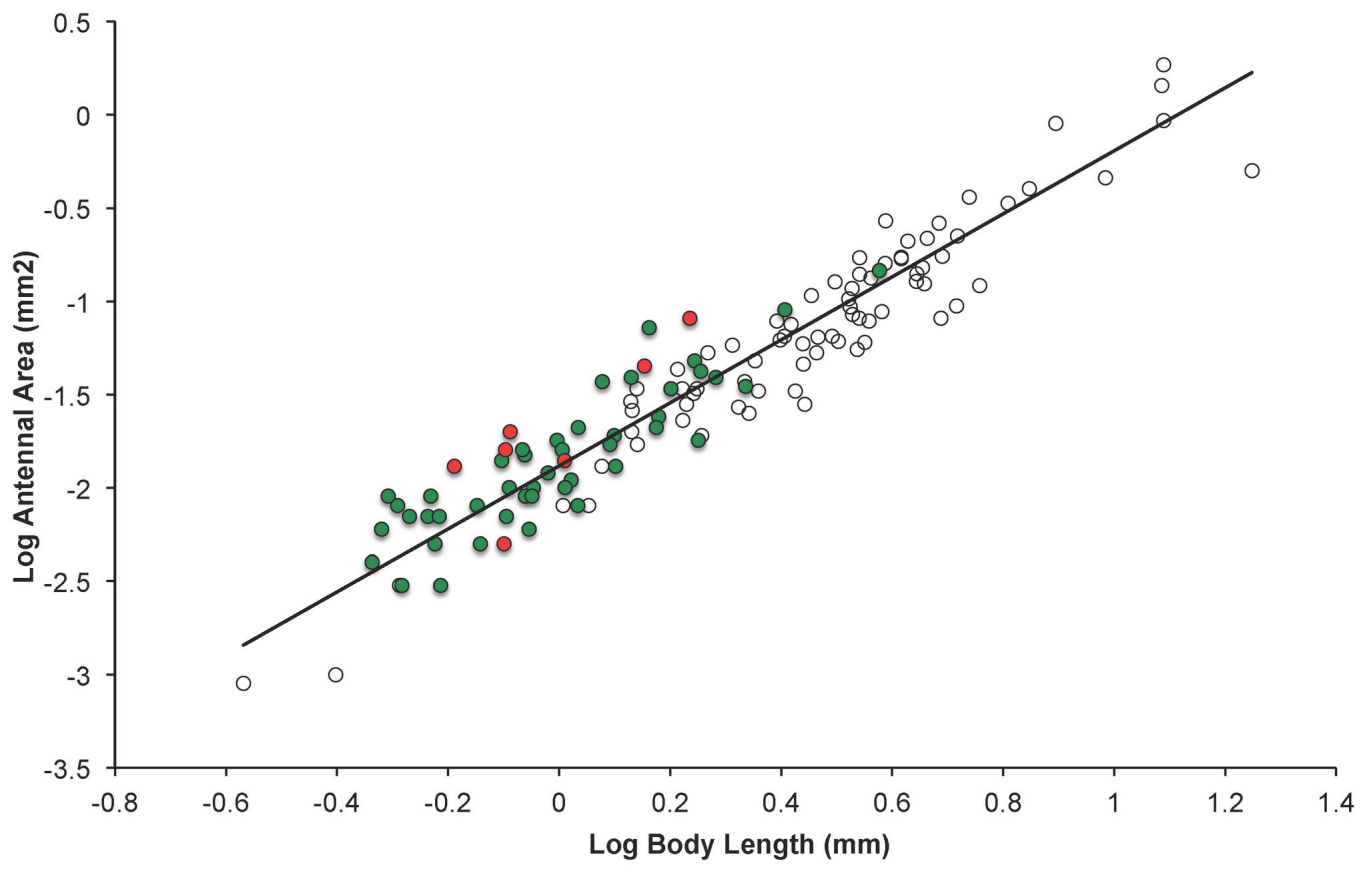
Antennal size and body size. Relationship between antennal size (log antennal area) and body size (log body length) for the 126 Chalcidoid species. The raw data are plotted with the phylogenetic generalized least squares regression line generated from the most recent chalcidoid phylogeny by Heraty et al. [20]. Species that parasitize Hemipteran hosts are indicated by filled circles with those parasitizing Sternorrhyncha in green and those that parasitize only other Hemipteran suborders in red. Note that species that parasitize Hemiptera are generally smaller than species that parasitize other orders, and that these species (particularly those that parasitize other Hemiptera) have relatively larger antennae for their body size (the majority of these species lie above the PGLS regression line).

Antennae size and body size were unsurprisingly highly correlated. Log antennal area scaled allometrically with log body length (PGLS Munro et al phylogeny: λ = 0.648, β = 1.700 (± 0.053 s.e.), R^2^ = 0.89; PGLS Heraty et al phylogeny: λ = 0.958, β = 1.689 (± 0.070 s.e.), R^2^ = 0.82; see also Figure 3). The slope value of around 1.7 was lower than the expected isometric scaling relationship of 2 for an areal measurement against a linear measurement, indicating that larger species had relatively smaller antennae.

### Phylogenetic signal

There was significant phylogenetic signal in most of the traits examined using both phylogenies (Tables 1, 2), indicating strongly that closely related species tend to have similar morphology and host preferences. Parasitoid behaviour itself is very strongly predicted by phylogeny – with non-parasitoid species being strongly clumped in certain clades, and likewise egg parasitoids being found in groups such as Myrmaridae and Trichogrammatidae. Likewise, the exploitation by parasitoids of a particular order of insects shows significant phylogenetic signal. In the case of parasitism of Hemiptera (both Sternorrhyncha and other Hemiptera), there is extremely strong phylogenetic constraint (the latter unsurprisingly given that most species parasitizing these other hemipterans are also egg parasitoids). Negative D values indicate that the trait is even more phylogenetically constrained than would be predicted under a Brownian motion model of evolution (see also Figures 1 and 2). Number of host species shows some phylogenetic signal (significantly so in the case of the Heraty et al. phylogeny). However, number of host orders or specialisation (specialist vs. generalist), and number of plant associates show little phylogenetic signal (λ values close to 0, D values close to 1), with no significant difference in trait distribution than would be expected from a random distribution of character traits across the tips of the phylogeny.

**Table 1:** Measures of phylogenetic signal in various traits of chalcidoid wasps using Munro et al.’s [18] phylogeny as the basis for analysis.

**Trait**	**λ**	***D***
Log body length	0.988*	
Residual log antennal area	0.529*	
Log number of host species	0.490	
Number of host orders	0	
Number of associated plant genera	0	
Parasitoid/non-parasitoid species		0.042*
Egg parasitoid		-0.129*
Specialist / generalist		0.935
Parasitize Hemiptera		-0.096*
*Parasitize Sternorrhyncha*		-0.042*
*Parasitize other Hemiptera*		-0.189*
Parasitize Diptera		0.687*
Parasitize Lepidoptera		0.521*
Parasitize Coleoptera		0.580*
Parasitize Hymenoptera		0.434*

For continuous variables the measure is Pagel’s λ [47,48], for discrete variables it is Fritz and Purvis’s [49] *D* metric. Traits that have statistically significant phylogenetic signal (i.e. significantly different from λ = 0 or *D* = 1, see methods for details) are indicated with an asterisk.

**Table 2:** Measures of phylogenetic signal in various traits of chalcidoid wasps using Heraty et al.’s [20] phylogeny as the basis for analysis.

**Trait**	**λ**	***D***
Log body length	1*	
Residual log antennal area	0.792*	
Log number of host species	0.644*	
Number of host orders	0.346	
Number of associated plant genera	0.143	
Parasitoid/non-parasitoid species		-1.900*
Egg parasitoid		-1.540*
Specialist / generalist		0.963
Parasitize Hemiptera		-1.854*
*Parasitize Sternorrhyncha*		-1.831*
*Parasitize other Hemiptera*		-2.157*
Parasitize Diptera		0.447*
Parasitize Lepidoptera		0.027*
Parasitize Coleoptera		- 0.075*
Parasitize Hymenoptera		-0.223*

### Relationship of body size to behavioural traits

We identified a number of traits that provide better predictors of body length than simply the null (intercept-only) model, as ascertained through comparison of AIC scores (see Tables 3, 4). Although there was no evidence that species with a greater number of host species had any differences in body size, there were weak indications from the Munro et al. phylogeny that specialist species and species that parasitize fewer orders are larger in size. These models had poor predictive value and were not much better than the null model (being on 1.2-2.4 times more likely), and there was no support for these models using the Heraty et al. phylogeny. Species associated with a greater number of plant genera were smaller in size, although the effect is small and the model was only better than the null model in the analysis with the Munro et al. phylogeny. Egg parasitoids tend to be smaller than other chalcidoids, but the effect is again small and only (just) appears as a better model than the null model in the analysis using the Heraty et al. phylogeny

**Table 3:** Single parameter models predicting body size (log body length) in chalcidoid species calculated from PGLS analysis, using Munro et al.’s [18] phylogeny as the basis for analysis.

**Trait**	**Λ**	**β (±s.e.)**	**R^2^**	**AIC**	**ER**
Log number of host species	0.990	-0.026 (±0.024)	<0.01	15.05	-
Number of host orders	0.992	-0.019 (±0.012)	0.02	13.72	1.21
Number of associated plant genera	0.988	-0.003 (±0.001)	0.04	11.18	4.29
Parasitoid (0)/non-parasitoid (1)	0.988	-0.056 (±0.090)	<0.01	15.71	-
Egg parasitoid (1)/other(0)	0.987	-0.035 (±0.075)	<0.01	15.87	-
Specialist (1)/ generalist (0)	0.994	0.076 (±0.037)	0.03	12.38	2.35
Parasitize Hemiptera	0.953	-0.326 (±0.054)	0.23	-12.76	676711
*Parasitize Sternorrhyncha*	*0.949*	*-0.342 (±0.053)*	*0.25*	*-16.40*	*2394300*
*Parasitize other Hemiptera*	*0.989*	*0.059 (±0.095)*	*<0.01*	*15.71*	*-*
Parasitize Diptera	0.988	0.005 (±0.046)	<0.01	16.08	-
Parasitize Lepidoptera	0.989	-0.036 (±0.044)	<0.01	15.44	-
Parasitize Coleoptera	0.979	0.128 (±0.048)	0.05	9.56	9.64
Parasitize Hymenoptera	0.992	-0.004 (±0.042)	<0.01	16.08	-
Null model (intercept only)	0.988	-	0	14.09	1

In cases where the AIC for the model is better (lower) than that for the null model, the evidence ratio (a measure of how much more likely the given model is than the null model) is presented. λ values are the maximum likelihood values for the entire model (both response and predictor variables)

**Table 4:** Single parameter models predicting body size (log body length) in chalcidoid species calculated from PGLS analysis, using Results using Heraty et al.’s [20] phylogeny as the basis for analysis.

**Trait**	**λ**	**β (±s.e.)**	**R^2^**	**AIC**	**ER**
Log number of host species	1	-0.017 (±0.023)	<0.01	-30.33	-
Number of host orders	1	-0.009 (±0.013)	<0.01	-30.25	-
Number of associated plant genera	1	-0.001 (±0.001)	0.01	-31.33	-
Parasitoid (0)/non-parasitoid (1)	1	0.023 (±0.078)	<0.01	-29.86	-
Egg parasitoid (1)/other(0)	1	-0.104 (±0.064)	0.02	-32.45	1.40
Specialist (1)/ generalist (0)	1	0.014 (±0.039)	<0.01	-29.91	-
Parasitize Hemiptera	1	-0.216 (±0.051)	0.13	-49.92	1949
*Parasitize Sternorrhyncha*	1	*-0.225 (±0.051)*	*0.14*	*-48.23*	*3752*
*Parasitize other Hemiptera*	1	*-0.026 (±0.081)*	*<0.01*	*-29.87*	*-*
Parasitize Diptera	1	0.013 (±0.046)	<0.01	-29.85	-
Parasitize Lepidoptera	1	-0.036 (±0.043)	0.01	-30.48	-
Parasitize Coleoptera	1	0.098 (±0.044)	0.04	-34.59	4.10
Parasitize Hymenoptera	1	0.043 (±0.041)	0.01	-30.88	-
Null model (intercept only)	1	-	0	-31.77	1

Of the stronger models, we found that species that parasitize Coleopteran hosts tend to be larger in size, whilst species that parasitize Hemipteran hosts tend to be smaller in size. More specifically, this effect is demonstrated for species parasitizing Sternorrhyncha (aphids, whiteflies and scale insects), but not the other Hemipteran suborders. These sternorrhynchan models are particularly strongly supported compared with the null model (the evidence ratio suggests over 2 million times more likely in the case of the Munro et al. phylogeny), and may explain as much as 25% of the variation in body size in these chalcidoid species (see also Figure 3).

### Relationship of antennal size to behavioural traits

**Figure 4:**
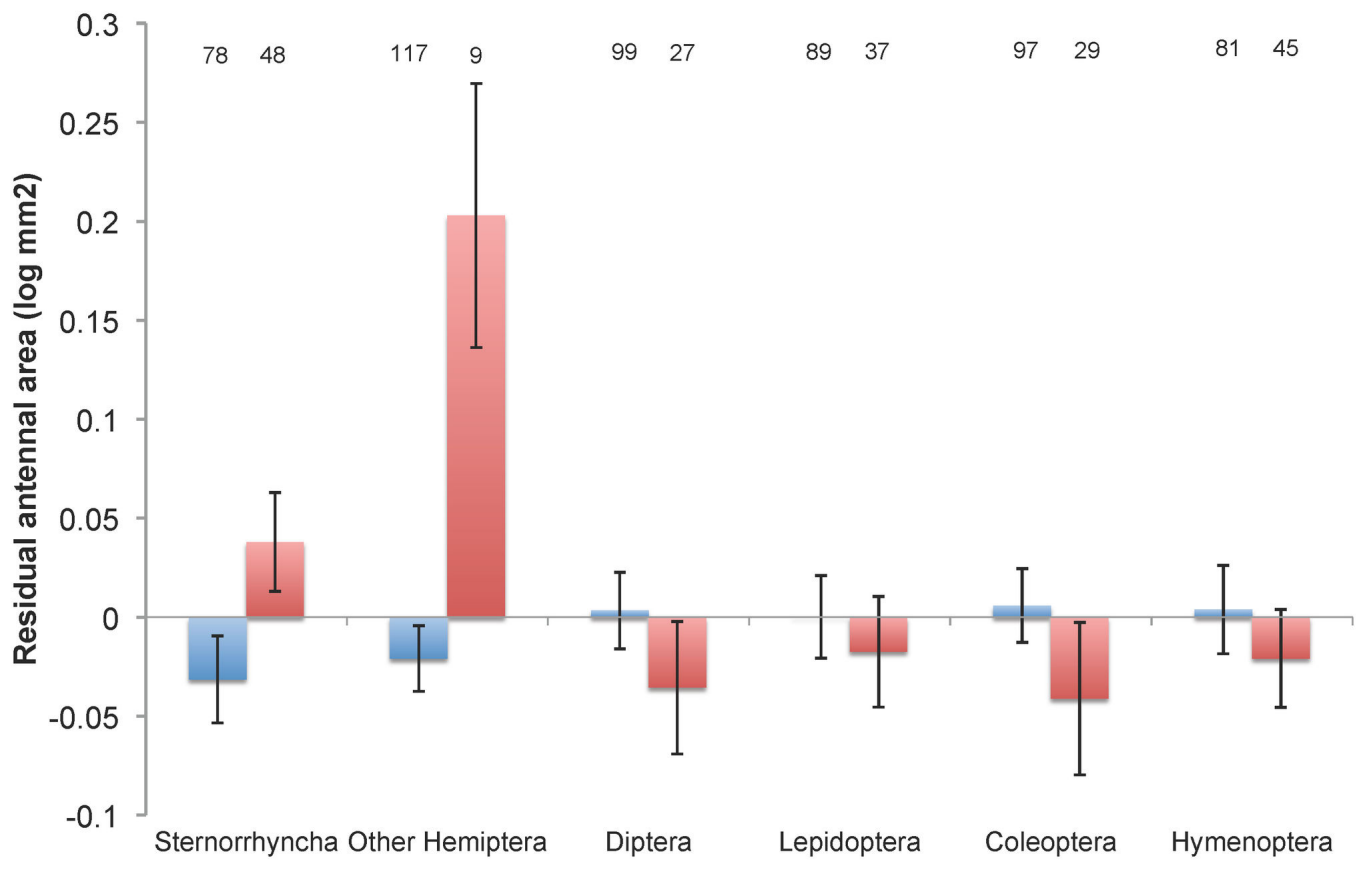
Antennal size and host use. Mean (±s.e.) residual antennal size for species of Chalcidoidea that do (red bars) and do not (blue bars) parasitize orders (and sub-orders) of insects. Group sample sizes are indicated above bar.

No measure of host breadth or specialisation predicted variation in antennal size (in models including, and hence controlling for, body size). Nor was number of plant associates a better predictor than the intercept-only model (Tables 5, 6). There was only one set of models predicting variation in relative antennal size that were convincingly better than the null models: species that parasitize Hemipteran hosts tend to have larger antennae (Figure 4). The effect was more weakly apparent in the parasitization of Sternorrhyncha, and only so in the analysis using the Munro et al. phylogeny. However, the effect was stronger in species that parasitize other Hemipteran hosts (evidence ratio up to 10 in the case of the analysis using the Heraty et al. phylogeny). By contrast, there was no strong support for models linking parasitism of other host orders and antennal size (Tables 5, 6; Figure 4). There was a suggestion using the Heraty et al. phylogeny of a slightly better model indicating that egg parasitoids have relatively larger antennae (only 1.4 times better than the null model). We consider this to be a result of the fact that most species that parasitize the Hemipteran suborders other than Sternorrhyncha are egg parasitoids. When we considered egg parasitism and parasitization of other Hemiptera in the same analysis (i.e. included both as predictors) the indication of larger antennae in the Hemipteran parasites remained (b = 0.152 ± 0.067 s.e.), but there was no clear indication of larger antennae in egg parasitoids generally (b = 0.052 ± 0.052 s.e.).

**Table 5:** Predictor variables from models examining variation in antennae size (log antennal area) among chalcidoid species, calculated from PGLS analysis using Munro et al.’s [18] phylogeny as the basis for analysis. All models include body size (log body length).

**Trait**	**λ**	**β (±s.e.)**	**R^2^**	**AIC**	**ER**
Log number of host species	0.641	0.010 (±0.018)	0.89	-78.01	-
Number of host orders	0.644	0.007 (±0.011)	0.89	-78.13	-
Number of associated plant genera	0.625	0.001 (±0.001)	0.89	-78.68	-
Parasitoid (0)/non-parasitoid (1)	0.635	-0.053 (±0.058)	0.89	-78.61	-
Egg parasitoid (1)/other(0)	0.647	0.047 (±0.052)	0.89	-78.60	-
Specialist (1)/ generalist (0)	0.650	0.027 (±0.034)	0.89	-78.41	-
Parasitize Hemiptera	0.647	0.132 (±0.046)	0.90	-85.71	19.70
*Parasitize Sternorrhyncha*	*0.620*	*0.088 (±0.047)*	*0.90*	*-81.29*	*2.16*
*Parasitize other Hemiptera*	*0.579*	*0.175 (±0.080)*	*0.90*	*-82.33*	*3.63*
Parasitize Diptera	0.643	-0.011 (±0.037)	0.89	-77.84	-
Parasitize Lepidoptera	0.648	-0.001 (±0.034)	0.89	-77.75	-
Parasitize Coleoptera	0.645	-0.008 (±0.039)	0.89	-77.80	-
Parasitize Hymenoptera	0.647	0.004 (±0.034)	0.89	-77.77	-
Null model (Log body length only)	0.648	1.700 (±0.053)	0.89	-79.75	1

All models include body size (log body length).

a Results using Munro et al. [18] phylogeny

**Table 6:** Predictor variables from models examining variation in antennae size (log antennal area) among chalcidoid species, calculated from PGLS analysis using Heraty et al.’s [20] phylogeny as the basis for analysis.

**Trait**	**λ**	**β (±s.e.)**	**R^2^**	**AIC**	**ER**
Log number of host species	0.956	0.007 (±0.018)	0.82	-83.68	-
Number of host orders	0.957	0.003 (±0.011)	0.82	-83.62	-
Number of associated plant genera	0.955	0.001 (±0.001)	0.82	-83.85	-
Parasitoid (0)/non-parasitoid (1)	0.952	-0.057 (±0.063)	0.82	-84.38	-
Egg parasitoid (1)/other(0)	0.973	0.084 (±0.051)	0.82	-86.22	1.40
Specialist (1)/ generalist (0)	0.954	0.020 (±0.032)	0.82	-83.95	-
Parasitize Hemiptera	0.918	0.143 (±0.045)	0.84	-93.12	44.04
*Parasitize Sternorrhyncha*	*0.943*	*0.066 (±0.047)*	*0.83*	*-85.53*	*-*
*Parasitize other Hemiptera*	*0.915*	*0.173 (±0.065)*	*0.84*	*-90.34*	*10.97*
Parasitize Diptera	0.964	-0.031 (±0.036)	0.82	-84.31	-
Parasitize Lepidoptera	0.958	-0.010 (±0.035)	0.82	-83.63	-
Parasitize Coleoptera	0.952	-0.006 (±0.038)	0.82	-83.58	-
Parasitize Hymenoptera	0.959	-0.011 (±0.034)	0.82	-83.66	-
Null model (Log body length only)	0.958	1.689 (±0.070)	0.82	-85.55	1

All models include body size (log body length).

## Discussion

Recent advances in our knowledge of the phylogeny of the Chalcidoidea provide enormous opportunities to investigate questions about the evolution of parasitoids in a broad comparative context. Previous phylogenetic comparative studies have focused on aspects of life-history evolution and the relationship of life-history strategies to types of host parasitism [10,53-57]. Here we provide evidence that aspects of host use are associated with difference in body size and antennal size in the parasitic Chalcidoidea.

The strongest relationship was that species that parasitize Sternorrhyncha tend to be smaller, and those that parasitize Hemiptera as a whole (and in particular suborders other than Sternorrhyncha) have relatively large antennae (for their body size). The association with body size in Sternorrhyncha-parasitizing species is much stronger than any of the other relationships that we examined – with possibly as much as a quarter of the interspecific variation in chalcidoid body size being explained by whether the species parasitize these sessile hemipterans or not. The relationship may reflect the fact that many Sternorrhyncha are small in size, and parasitoid body size is associated with host size generally [11].

Because we examined only female wasps, we need also to consider the possibility of sex differences in body size influencing our results. Some chalcidoids, specifically in the Coccophaginae (which in this study is represented by *Encarsia, Coccobius* and *Coccophagus*), exhibit heteronomy, where females develop in hemipteran hosts, while males develop as parasitoids of other orders of insects, including Hymenoptera, and even as parasitoids of females of the same species [58]. These differences can create sexual dimorphism in body size [58], although with females being larger, not smaller than males [59]. However, given Coccophaginae is monophyletic in both phylogenies, there appears only to be a single evolutionary origin for heteronomy in Chalcidoidea [18,20]. Consequently, with only a single evolutionary transition and all heteronomous species being closely related, the impact of this trait on our overall results in a phylogenetically controlled analysis should be minimal. Nevertheless, it would be interesting to compare levels of sexual dimorphism in Chalcidoidea in relation to host use and specialisation.

Of perhaps greater interest was our finding that species that parasitize Hemiptera typically have relatively larger antennae. The effect appeared to be more pronounced in species that parasitize Heteroptera and Auchenorrhyncha, and less obvious in species parasitizing Sternorrhyncha. In insects generally, larger antennae are typically associated with greater numbers of olfactory receptors [14] resulting in greater sensitivity and acuity to chemical signals [60,61]. Since chemical signals are the primary means by which chalcidoid wasps locate their hosts, including hemipterans [62,63], this may indicate some feature of hemipteran chemical communication, such as reduced amount of chemicals produced, or more volatile chemicals produced [64], that selects for greater sensitivity and hence antennal size in the parasitoid. In particular, parasitoids of true bugs rely on long-range kairomones from nontarget instars (most are egg parasitoids) and host-plant synomones induced by host feeding [65]. However, broad-scale comparative evidence of associations between sensilla number or density and antennal size in Chalcidoidea are lacking (a 2007 review [13] identifies such information for only a dozen species), and the available evidence does not necessarily support a link between antennal size and sensilla number. For example some pteromalid species have short antennae but dense patches of sensillae, and females exhibit greater density of sensillae despite no appreciable difference in antenna length [66]. Consequently, until a comparative meta-analysis of antennal size and sensilla number (or sensory capability) is available, any chemosensory explanation for the large antennae of Hemipteran-exploiting parasitoids must necessarily be speculative.

Alternatively, larger antennae may offer a degree of safety from counter-attacks by potential hosts: large antennae may allow the wasp to gather information about host suitability, while keeping its body at a safe distance. The strongest effect on antennal length was among chalcidoids that parasitize Heteroptera and Auchenorrhyncha, suborders of Hemiptera whose members are often predatory, and provide parental care of eggs (many species parasitizing these orders are egg parasitoids) [67,68]. Species among the relatively defenceless Sternorrhyncha are also capable of defensive behaviours, such as evasive manoeuvres and alarm pheromone-induced attack behaviours (for review see ref[69].). Additionally, some aphid species produce sticky compounds that may entrap parasitoids, and some parasitoids of Hemiptera avoid placing their antennae directly on the host, with consequent differences in the fine morphology of the antennae of such species [13].

We found no evidence for an association between breadth of hosts and antennal size, and in particular that more specialist species have larger antennae. This pattern is not consistent with Chapman’s [14] prediction, although that referred specifically to the number of antennal sensilla, not antennal size *per se*.

Our analysis provides weak evidence that species that parasitize a smaller number of orders, or that are specialists (i.e. parasitize fewer than 10 species in a single order) are larger in size. However, given the small effect size, and the models not being appreciably better than the null model, we hesitate drawing strong biological inferences. In a previous study, Mayhew and Blackburn [53] found no significant difference in body size between koinobiont (where hosts continue to develop after parasitism) and idiobiont (where hosts are immobilised after parasitism) parasitoids. The former are generally specialists compared with the latter [70,71]. Traynor and Mayhew [10] reported that egg parasitoids were significantly smaller in size than other parasitoids, and our analysis did reveal a similar pattern, but with only one of the phylogenies suggesting a marginally better model than the null intercept only model.

Smaller species of parasitoids tended to be associated with exploiting a greater number of host plant genera. The effect is still not strong, although AIC values suggest this model *is* appreciably better than the null model in the analysis using the Munro et al. phylogeny. If exploiting a broader range of hosts is more costly through exposure to a wider range of defensive compounds [22], then smaller body size might be adaptive. For example, by reducing body size, species may speed up developmental times, and thus limit exposure to these defensive compounds [72]. Clearly, though, the extent to which this may be having an effect depends on whether the hosts are actively using plant defensive compounds as a defence against parasitoids.

The phylogenetic constraint on our traits of interest varied, and the strength of phylogenetic signal depended on the phylogeny used as the basis of analysis. Body size and antenna size exhibited significant phylogenetic signal in both analyses, particularly strongly in the former. Body size and other morphological variables exhibit strong phylogenetic dependence across a wide range of animal taxa [73,74]. The signal in antennal size was stronger in the analysis using the Heraty et al. phylogeny, which is perhaps not surprising since this phylogeny was based in part on morphological characteristics, including antennal structure, so there may be an inevitable tendency for species with similar antennal morphology to be grouped together on the phylogeny. By contrast, measures of host and plant breadth/specificity exhibit weaker phylogenetic signal – indeed there are numerous examples in our data set of extreme variation in host specificity among species within the same genus. For example, *Aprostocetus plesispae* Ferrière is associated with a single species of chrysomelid beetle, whilst *Aprostocetus minutus* (Howard) parasitizes at least 37 species within the Hemiptera, Coleoptera and Neuroptera. Our results are consistent with an earlier study of aphid parasitoids that found no relationship between taxonomy and host range breadth [75]. Lack of phylogenetic signal in host specificity may be widespread in host-parasite systems. For example, a recent analysis found generally weak phylogenetic signal in host specificity in parasitic fleas [76], although unlike here, the signal was stronger when considered across broad taxonomic and geographic (continental) scales.

In general, there was strong phylogenetic signal in parasitoid/non-parasitoid behaviour itself, unsurprisingly as there are well known groups within Chalcidoidea that are predominantly non-parasitic, such as the fig wasps within the Agaonidae. Egg parasitism is also a highly phylogenetically constrained trait, as previously identified in the phylogenetic analyses by Munro et al. and Heraty et al. [18,20]. But the identity of host orders also exhibits significant phylogenetic signal, particularly strongly for species that parasitize the constituent groups of Hemiptera (Sternorrhyncha and Heteroptera/Auchenorrhyncha) – where the pattern is even more conserved than would be predicted from a Brownian motion model of evolution. In their analysis [20] Heraty et al. identified strong phylogenetic patterns in parasitism of Sternorrhyncha, with far fewer origins of the trait than had previously been identified in the Munro et al. [18] analysis (see also Figures 1 and 2, where the Munro et al. phylogeny suggests a minimum of 10 separate origins of this trait, compared to only 5 or 6 for the Heraty et al. phylogeny). In addition to Sternnorrhyncha, parasitism of Heteroptera and Auchenorrhyncha is clearly phylogenetically clumped – notably in the Myrmaridae (including *Gonatocerus*) and Trichogrammatidae. It is worthwhile noting that a clear association between antennal morphology and this trait was still found even after controlling for the confounding effects of this strong phylogenetic signal.

Although parasitization of the Hemipteran suborders showed the strongest phylogenetic signal, significant signal was also found in the traits for parasitizing all the other insect orders. These results suggest that, whilst switches between host specialists and generalists is extremely common in the evolutionary history of chalcidoid parasitoids, switching between hosts from different orders is not so common among specialists – perhaps understandably as this may involve the evolution of entirely different morphological, behavioural and sensory adaptations. Further evidence for the links between host use and parasitoid phylogeny can be found in a recent analysis of Eucharitidae [77]. Members of this family parasitize ants and there is a significant overlap between host and parasitoid phylogeny.

Of course, any phylogenetic comparative analysis is necessarily constrained by the species examined. Even within these data there are species that appear to confound the general results. For example the trichogrammatid species *Prestwichia aquatica* Lubbock, which parasitizes Heteropteran water bugs (and water beetles), has actually among the smallest antennae relative to its body size of any of the species in our analysis. Obviously, many other factors drive body size and antennal morphology than considered here, but the power of comparative analyses lies in the ability to detect correlated evolution and provide more substantial support for general evolutionary hypothesis than can be derived from individual species comparisons or analyses of single clades.

## Supporting Information

Table S1 **Chalcidoid morphological and ecological data.**
(XLSX)
